# Machine learning models for predicting in-hospital mortality in patient with sepsis: Analysis of vital sign dynamics

**DOI:** 10.3389/fmed.2022.964667

**Published:** 2022-10-20

**Authors:** Chi-Yung Cheng, Chia-Te Kung, Fu-Cheng Chen, I-Min Chiu, Chun-Hung Richard Lin, Chun-Chieh Chu, Chien Feng Kung, Chih-Min Su

**Affiliations:** ^1^Department of Computer Science and Engineering, National Sun Yat-sen University, Kaohsiung, Taiwan; ^2^Department of Emergency Medicine, Kaohsiung Chang Gung Memorial Hospital, Chang Gung University College of Medicine, Kaohsiung, Taiwan; ^3^Graduate Institute and Department of Intelligent Commerce, National Kaohsiung University of Science and Technology, Kaohsiung, Taiwan

**Keywords:** sepsis, dynamic vital sign, mortality, prediction model, machine learning model

## Abstract

**Purpose:**

To build machine learning models for predicting the risk of in-hospital death in patients with sepsis within 48 h, using only dynamic changes in the patient's vital signs.

**Methods:**

This retrospective observational cohort study enrolled septic patients from five emergency departments (ED) in Taiwan. We adopted seven variables, i.e., age, sex, systolic blood pressure, diastolic blood pressure, heart rate, respiratory rate, and body temperature.

**Results:**

Among all 353,253 visits, after excluding 159,607 visits (45%), the study group consisted of 193,646 ED visits. With a leading time of 6 h, the convolutional neural networks (CNNs), long short-term memory (LSTM), and random forest (RF) had accuracy rates of 0.905, 0.817, and 0.835, respectively, and the area under the receiver operating characteristic curve (AUC) was 0.840, 0.761, and 0.770, respectively. With a leading time of 48 h, the CNN, LSTM, and RF achieved accuracy rates of 0.828, 0759, and 0.805, respectively, and an AUC of 0.811, 0.734, and 0.776, respectively.

**Conclusion:**

By analyzing dynamic vital sign data, machine learning models can predict mortality in septic patients within 6 to 48 h of admission. The performance of the testing models is more accurate if the lead time is closer to the event.

## Highlights

– By analyzing dynamic vital sign data, machine learning models can predict mortality in septic patients within 6–48 h of admission.
– The performance of the machine learning models is more accurate if the lead time is closer to the event.

## Introduction

Sepsis is the presence of an acute infection and new organ dysfunction. It can be life-threatening if not recognized and treated promptly (1). Despite advanced care, previous studies have demonstrated that sepsis remains a significant burden worldwide and is the most common cause of in-hospital deaths (2–4). Although outcomes have improved in recent decades, mortality remains high at approximately 25–30% (5). Furthermore, septic shock is associated with an even higher mortality rate of ~40–50% (6). For patients at critical risk, increased awareness, aggressive treatment, and broad-spectrum empiric antibiotics significantly decrease the mortality risk (7). It is therefore imperative to rapidly and accurately stratify patients with sepsis and high in-hospital mortality.

In recent decades, medical artificial intelligence (AI) has been used to achieve clinical diagnoses and suggest treatments. A few examples where AI has shown promise for clinical diagnoses include diabetic retinopathy screening (8), skin lesion classification (9), and assist in detection of abdominal free fluid during focused assessment with sonography (10). In combination with machine learning algorithms and electronic health records (EHRs), clinical data sources enable us to rapidly generate prediction models and predict clinical outcomes. For instance, an AI model has been used to predict the mortality of patients diagnosed with COVID-19 (11), outcomes in trauma patients (12), and neurological outcomes of out-of-hospital patients after a cardiac arrest (13).

Machine learning methods can predict in-hospital mortality in sepsis patients in an intensive care unit (ICU) (14). At the time of sepsis onset, Barton et al. demonstrated that a machine learning algorithm with gradient-boosted trees increases the sensitivity and specificity of predicting sepsis occurrence over the commonly used systemic inflammatory response syndrome (SIRS), modified early warning score (MEWS), sequential organ failure assessment (SOFA), and quick sequential organ failure assessment (qSOFA) scoring systems (15). In addition, machine learning algorithms can predict the occurrence of severe sepsis and septic shock (14, 16). For predicting in-hospital mortality of ED patients with sepsis, Taylor et al. found that a machine learning approach outperformed existing clinical decision rules (17).

However, most previous prediction models for mortality require a large number of variables, including the underlying disease, laboratory data, and clinical parameters. The aim of our study was to build ML models for predicting the risk of in-hospital death in patients with sepsis within 48 h, using only dynamic changes in the vital sign.

## Methods

### Study population and extraction samples

This is a retrospective observational cohort study conducted from January 1, 2006 to December 31, 2017. The study was approved by the IRB Review Board of the Chang Gung Medical Foundation (IRB number: 201801713B0; approved on 28 January 2019) in accordance with the ethical guidelines of the 1975 Declaration of Helsinki. Informed consent was not required owing to the retrospective nature of the study.

We used data provided by the Chang Gung Medical Center, including five EDs that belonged to a single healthcare system and were geographically dispersed nationwide in Taiwan. Sepsis patients were extracted from the electronic database records of the Chang Gung Medical Center under the following conditions: (1) The age of the patient was over 17 years, (2) blood culture was obtained, and (3) antibiotics were prescribed in medical order. Sepsis patients were defined according to the Third International Consensus Definition of Sepsis (Sepsis-3) definition, that was an acute change in Sequential [Sepsis-related] Organ Failure Assessment (SOFA) score of 2 points or more consequent to the infection (1).

We excluded patients who had an out-of-hospital cardiac arrest because their high mortality rates could falsely affect the performance of the prediction models. Besides, we also excluded patients for the following reasons: (1) the length of the hospital stay was >3 days (2) the patients recorded less than three times when they stay in hospital, and (3) patients with incorrect data and format. The selection process and sample numbers were listed in the Supplementary Table 1.

The outcome was divided into two results: positive instances in which patients died in the hospital and negative instances in which patients survived. After cleaning problematic data such as those containing less than one record for every variable, and those having an error in terms of format, the number of positive instances was 19,434, and the other negative instances numbered 194,646. Supplementary Table 1 presents the detailed sample selection process. We found that the number of negative instances was 10-times greater than the number of positive instances. The number of surviving patients was 16-times the number of deceased patients. To resolve the imbalanced sample problems, we used random sampling in negative instances to balance the number of positive and negative instances. We use python programs which the system provides the function random choice(). The function random choice() can choose the instances from the negative instances randomly and the amount of the negative instances we requested.

### Feature selection and data processing

To construct a mortality prediction tool, we adopted five vital signs: systolic blood pressure (SBP), diastolic blood pressure (DBP), heart rate (HR), respiratory rate (RR), and body temperature (BP). Patient age and sex were also included. Vital signs were selected as objective predictor variables because they are routinely and frequently collected, regardless of the clinical situation, and the values are rarely affected by the examiner.

There were two different types of outcomes in this research. For these two outcomes, we extracted negative instances from all subjects who survived and positive instances from all subjects who died in the hospital. The data for up to 6–48 h prior to death were extracted as a positive instance, and the data for up to 6–48 h prior to survival or discharge were extracted as a negative instance, as shown in Supplementary Figure 1.

We mainly focused on four different lead times (k = 6, 12, 24, and 48) prior to the results, and we used machine learning and developed four models to predict the results k hours in advance.

The subsequent cleaning process ensured that the electronic data were ready for analysis and did not contain any errors. First, we removed problematic records. Second, to resolve the problems of missing data, the measured value of the vital sign variable was forward-filled following an initial measurement until the next available measurement. The choice was based on the clinical insight that measurements are taken more frequently during times of hemodynamic instability and less frequently when the patient appears stable. Third, the records were converted into z-scores using the computed means and standard deviations. These vital sign variables were normalized to [0, 1] for comparison. Fourth, the vital sign normalized values are divided into 255, according to the degree of the divided results, converted into grayscale, and transformed into an image for each patient.

### Machine learning

To build appropriate models and develop an early warning system (EWS), we set the training, validation, and testing sets to a ratio of 6:2:2. The early warning system models developed using convolutional neural networks (CNN) was labeled as EWS_C, using long short-term memory (LSTM) was labeled as EWS_L, and using random forest (RF) was labeled as EWS_R, respectively.

CNNs are the most mature tools in graphical process in machine learning. It is a class of deep feed-forward artificial neural networks, and a CNN architecture is formed by a stack of distinct layers that transform the input volume into an output volume through a differentiable function (18). A few distinct types of layers contain a convolutional layer, a pooling layer, an activation layer, a fully connected layer, and a loss layer, the conceptual architecture of which is illustrated in Supplementary Figure 2 and Supplementary Table 2.

LSTM is effective for capturing the underlying temporal structures in time-series data. It consists of the following three gates: forget, input, and output gates. These three gates interact to control the flow of information. LSTM builds memory by feeding the previous hidden state as additional input in the subsequent step. This makes the model particularly suitable for modeling dynamics in vital sign data, which has a strong statistical dependency between medical events over the time intervals. LSTM enables the network to maintain the previous information of the hidden states as internal memory (19). The network architecture of the LSTMs are listed in Supplementary Table 3. Parameters of Random Forest model.

RF is an efficient, multi-class approach that is able to handle large attribute spaces, and has been widely used in several domains including real-time face recognition and bioinformatics (20). RF is an ensemble method used to construct many decision trees that are applied in the classification of a new instance based on a majority vote. Each decision tree node uses a subset of attributes that are randomly selected from the entire original set of attributes. The RF model parameters are listed in Supplementary Table 4.

The flow of the applied research method is described in the previous section. Supplementary Figure 3 presents the overall research flow diagram.

For the reliability and stability of our models in this research, our research adopted two validation methods. The first validation, we use k-fold cross-validation methods considered our sample sizes, we adopted k-fold = 5 for model tuning and yield a satisfying generalization performance. In k-fold cross-validation, we randomly spilt the training dataset in k folds without replacement, where k-1 folds are used for the models training and one folds is used for testing. This procedure is repeated k times and we obtain k models and performance estimates. Then, we calculate the average and 95% confidence interval performance of the models based on the different, independent folds to obtain a performance estimate that is less sensitive to the sub-partitioning of the training data. We listed the cross-validation results of EWS_C, EWS_L, and EWS_R in the Supplementary Tables 8–10, respectively.

The second validation part was reserved the data of 2017 as extra validation part. The EWS_C, EWS_L, and EWR_R were validated the data of 2017, and the results were listed in the validation part in the Supplementary Tables 5–7, respectively. From the 1-year clinical validation, our research results were more reliable and stability.

### Statistical analysis

To accurately build the early warning system model, we compared it with other standard machine learning algorithms, i.e., CNN, LSTM, and RF. We call these models EWS_C, EWS_L, and EWS_R, respectively. The model performance was assessed based on discrimination using the precision, recall, accuracy, receiver operating characteristic (ROC) curve, and derived area under the ROC curve (AUC).

## Results

### Dataset statistics

The symbols in this research included 28,530 positive instances and 194,646 negative instances. We also excluded (1) records from <72 h after admission to the ED, (2) cases with fewer than three records, and (3) incorrect data or an improper format. To summarize, k = 6, 12, 24, and 48 h in the numbers of excluded and included samples are listed in Supplementary Table 1.

We extracted the vital signs of the patients in k (where k = 6, 12, 24, and 48 h) prior to the time of death as a positive instance. By contrast, we extracted the vital signs of the patient k (h) prior to the time of survival as a negative instance. The smaller the lead time before the result is, the larger the number of patient cases. For example, when k = 6 h, there are 19,434 positive instances and 194,646 negative instances; in other cases, when k = 48 h, there are 17,123 positive instances and 125,102 negative instances.

### Mortality prediction performance

We compared the models among these three methods: CNNs, LSTMs, and RF. To distinguish which model is more accurate and reliable and help doctors make decisions, we also compared four different lead time models, i.e., 6, 12, 24, and 48 h models.

**Figure 1:**
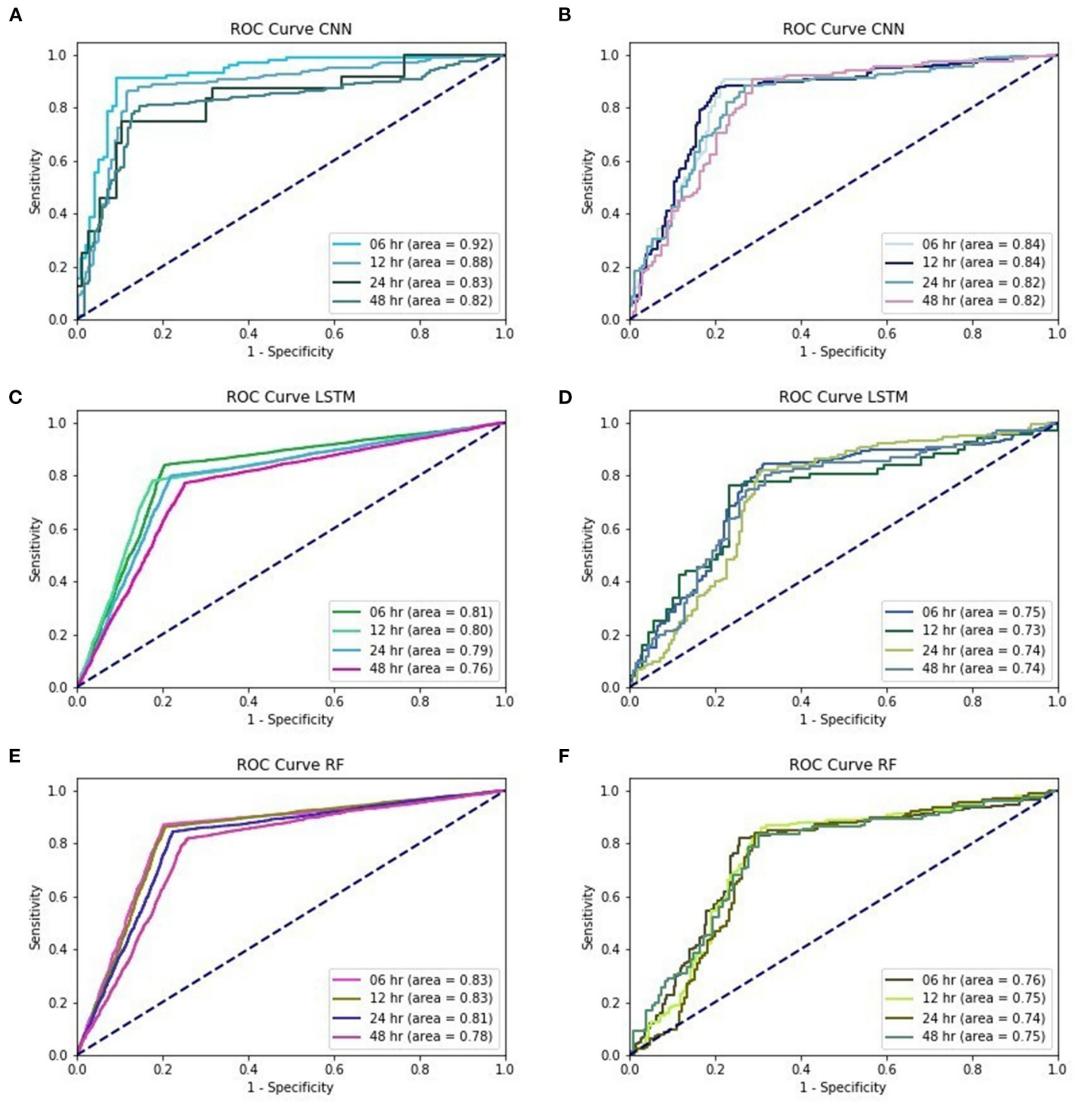
**(A)** training performances of ROC curve in EWS_C, **(B)** testing performances of ROC curve in EWS_C, **(C)** training performances of ROC curve in EWS_L, **(D)** testing performances of ROC curve in EWS_L, **(E)** training performances of ROC curve in EWS_R, **(F)** training performances of ROC curve in EWS_R.

In the first part, we used the CNN-based algorithm under different lead-time models for EWS_C. With EWS_C, the precision, sensitivity, and accuracy values of the training and validation models are summarized in Supplementary Table 5. In the validation model, we also provide AUC_COV and the confidence interval (CI) under different lead-time models. In addition, we provide the ROC curve of EWS_C in Figure 1A.

According to the validation models for EWS_C, the records show that the precision among these four different lead time models is above 0.85. The sensitivity was above 0.75 for EWS_C6 and EWS_C12, and the sensitivities for EWS_C24 and EWS_C48 were above 0.7. In addition, the accuracy for EWS_C was >0.8. In Figure 1A, we found that the AUC in the EWS_C training model at 6 h was the largest, reaching 0.92. We also found that the other lead times (12, 24, and 48 h) and their AUC were all above 0.8. In the testing of the EWS_C model for all lead times, the ROC curves are all above 0.8 (Figure 1B). In Supplementary Table 6 and Figure 1C, we summarize the training and validation results. We found that the precision, sensitivity, and accuracy are ~0.8, 0.7, and 0.75. As shown in Figure 1D, the ROC curve for EWS_L testing was approximately 0.75. Supplementary Table 7 summarizes the results of the training and validation of EWS_R. We found that the validation of EWS_R had a precision of approximately 0.8, a sensitivity of ~0.7, and an AUC of nearly 0.77. According to Figure 1E, for the ROC curve of EWS_R in the training model, the area of all lead times was over 0.8. In the testing model, the area of all lead times was over 0.7 (Figure 1F).

**Figure 2:**
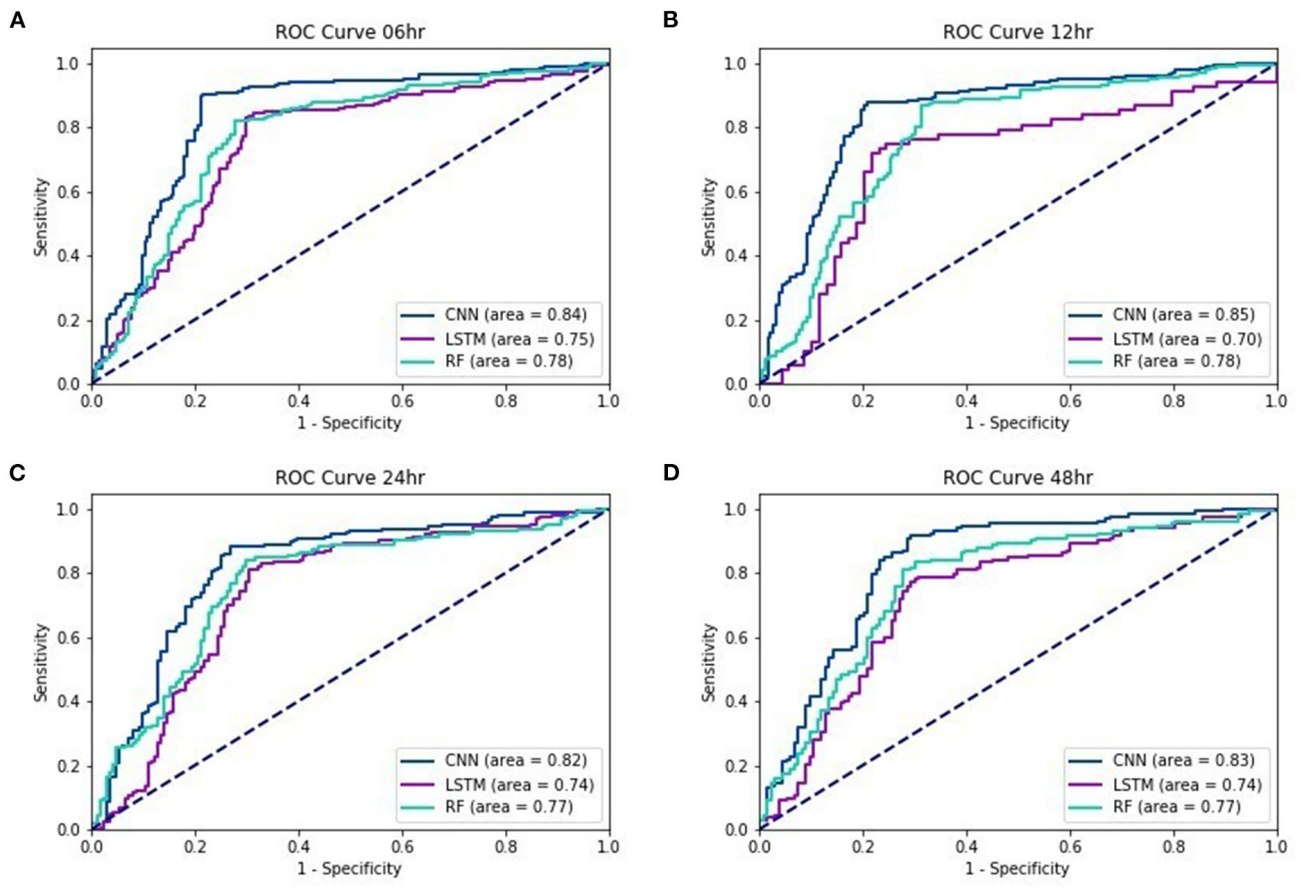
ROC curves of EWS_C, EWS_L, and EWS_R: **(A)** 6 **(B)** 12, **(C)** 24, and **(D)** 48 h lead times.

Figure 2 show the ROC curves of the three testing models for lead times of 6, 12, 24, and 48 h. Regardless of the lead time, we found that the AUC of EWS_C was the largest. For a 48-h lead time, the AUC was still over 0.8.

## Discussion

In our study, machine learning models were used to predict the mortality in septic patients 48 h prior to death. The AUC of the testing models for a 48-h lead time is 0.83, 0.74, and 0.77 with the EWS_C, EWS_L, and EWS_R models, respectively. In general, the performance of the testing models is more accurate if the lead time is closer to the event. The AUC of the testing models under a 6-h lead time could achieve values of 0.84, 0.75, and 0.78 for EWS_C, EWS_L, and EWS_R, respectively. For all lead times, we found that the AUC of EWS_C had the best model performance among the ML models, with an AUC within the range of 0.82–0.85.

A wide array of rule-based scoring systems was developed to assess the severity of illness and risk stratification. Examples frequently used as severity assessment tools in the ICU are the simplified acute physiology score (SAPS) II (21), acute physiology and chronic health evaluation (APACHE) III and IV scores (22, 23), and the SOFA score (24). As a major limitation of the above systems applied in the ED, they require information that is often not readily available during a patient's time in the ED. Therefore, EWSs were developed to detect patients at risk of deterioration and predict catastrophic events in an ED. For the general ED population and patients with respiratory distress, the NEWS achieves the highest accuracy in mortality prediction (25). For patients with infection or sepsis, the MEDS and MEWS were the most utilized methods of assessment. In general, the MEDS (AUC of 0.73–0.871) achieves a better accuracy than MEWS (AUC of 0.596–0.73) in predicting in-hospital mortality (26–30). Other prognostic scores frequently used in an ED include a rapid emergency medicine score and the qSOFA, with an AUC range of 0.62–0.80 and 0.58–0.76, respectively (31, 32).

These EWSs were created mostly based on physiological measurements and clinical observations, including vital signs, level of consciousness, laboratory data, and other metrics, depending on the selected modification tool. Thus, most of these scores are complex or disease-specific, leading to a poor early recognition of septic patients at risk of deterioration. Moreover, the differences between the observed and expected mortality may also be caused by inadequate diagnostic data, unreliable Glasgow coma scale (GCS) score assessment, regional differences, and changes in the effectiveness of therapy over time (33, 34).

With the progress and development of big data techniques, machine learning methods have attracted research attention in the past decade. Zhang et al. found that the least absolute shrinkage and selection operator technique achieves a good discrimination and calibration for mortality prediction in patients with severe sepsis (35). Using over 500 clinical variables, Taylor et al. demonstrated that the machine learning approach outperformed existing clinical decision rules, with the RF model performing better than the LR model in terms of discrimination (17). Considering a total of 587 features, including demographics, vital signs, and laboratory results, Giannini et al. showed that an RF classifier can predict the impending occurrence of severe sepsis and septic shock with a low sensitivity of 26% and high specificity of 98% (16). Misra et al. indicated that using clinical and administrative data, machine learning models can be applied to predict septic shock within the first 6 h of admission, with a sensitivity of 83.9% and a specificity of 88.1% based on RF (36). Utilizing nine features combined with vital signs, chief compliances, and the emergency severity index, Klug et al. concluded that the gradient boosting model shows a high predictive ability for screening patients at risk of early mortality using data available at the time of triage in the ED (37). However, most of the previous machine learning methods for predicting the prognosis of patients with sepsis required numerous variables, including laboratory results, GCS, and clinical parameters.

Several studies highlight the value of dynamic vital sign changes for building predictive models. A pilot study used physiomarkers to generate 52 highly ranked features and build an eXtreme Gradient Boost classifier that could predict post-liver transplant patients 12 h before developing sepsis (38). Another observational cohort study yielded a total of 60 features from physiomarkers, and revealed predict severe sepsis 8 h prior to the event in critically ill children (39). Van Wyk et al. found that using continuous physiological data alone to generate a total of 132 features, random forest classifier could discriminate sepsis 5 h before the onset (40). Using five physiological data streams including HR, RR, and BP (systolic, diastolic, and mean), Mohammed et al. developed a support vector machine (SVM) classifier for predict sepsis up to interval of 17.4 h before sepsis onset, with an average test accuracy, sensitivity, specificity, and area under the receiver operating characteristics curve of 0.83, 0.757, 0.902, and 0.781, respectively (41).

However, using only physiological data, previous studies mostly focused on predict sepsis event. By contrast, our study focused on predict mortality. We included only seven input parameters in our study, including age, sex, and vital signs (BT, SBP, DBP, HR, and RR), available from the moment of triage to any time during hospitalization. Using data available in the ED in real time, artificial intelligence can accurately predict mortality in septic patients 6–48 h prior to clinical recognition.

The proposed method has several advantages. First, vital signs had clear-cut values and were obtained through machine measurements, which reduced the expert judgment and limited variations in the healthcare providers. In addition, models that require hundreds of variables may lead to difficulty in encoding the databases and may have more missing values or data errors. Instead, we attempted to develop an uncomplicated model that requires simple input parameters that are routinely collected during daily practice. A simplified tool would be more easily implemented in resource-limited ED settings. Furthermore, the data used in our model were widely available in clinical practice. Lukaszewski et al. reported that neural networks can correctly predict patient outcomes of overt sepsis prior to clinical diagnosis with high sensitivity and selectivity (91.43 and 80.20%, respectively) (42). Because cytokines are not routinely measured, this tool is impractical in clinical practice. Instead, our study attempted to develop a simplified model with feasible and reliable input parameters that can be efficiently collected in place with limited medical resources.

Our study has several limitations. First, this was a retrospective study conducted in Taiwan. The sample was homogeneous and may have been subject to local practices, limiting its generalizability to other ethnicities. Second, we did not compare all available ML models and scoring systems, or their variations. There are hundreds of different ML models and variations; therefore, a comprehensive study is unfeasible. Application of the developed ML model to other datasets or populations requires a further clinical evaluation.

## Conclusion

This study contributes to clinical areas using machine learning in-hospital mortality prediction models for sepsis patients in the ED. By analyzing dynamic vital sign data, machine learning models can predict mortality in septic patients within 6–48 h of admission. The CNN achieves the best model performance in comparison to the LSTM and RF approaches. In general, the performance of the testing models is more accurate if the lead time is closer to the event.

## Data availability statement

The raw data supporting the conclusions of this article will be made available by the authors, without undue reservation.

## Ethics statement

The studies involving human participants were reviewed and approved by Chang Gung Medical Foundation. Written informed consent for participation was not required for this study in accordance with the national legislation and the institutional requirements.

## Author contributions

CK and C-MS conceived the study and assumed responsibility for the paper as a whole. I-MC and C-HL managed the data and including quality control. C-TK, F-CC, and C-CC provided statistical advice on the study design and analyzed the data. C-YC and C-TK chaired the data oversight committee. C-YC drafted the manuscript and all of the authors contributed substantially to its revision. All authors read and approved the final manuscript.

## Conflict of interest

The authors declare that the research was conducted in the absence of any commercial or financial relationships that could be construed as a potential conflict of interest.

## Publisher's note

All claims expressed in this article are solely those of the authors and do not necessarily represent those of their affiliated organizations, or those of the publisher, the editors and the reviewers. Any product that may be evaluated in this article, or claim that may be made by its manufacturer, is not guaranteed or endorsed by the publisher.
