# Supplementary material for: Machine learning models for predicting in-hospital mortality in patient with sepsis: Analysis of vital sign dynamics
Source: Front Med (Lausanne). 2022 Oct 20;9:964667. doi: 10.3389/fmed.2022.964667 (PMC9631306; doi:10.3389/fmed.2022.964667)

Supplemental data file

Supplemental table 1. Sample selection process

Supplemental table 2. Network architecture of CNN

Supplemental table 3. Network architecture of LSTM

Supplemental table 4. Parameters of Random Forest model

Supplemental table 5. Training and validation results of EWS_C

Supplemental table 6. Training and validation results of EWS_L

Supplemental table 7. Training and validation results of EWS_R

Supplemental table 8. Cross Validation results of EWS_C

Supplemental table 9. Cross Validation results of EWS_L

Supplemental table 10. Cross Validation results of EWS_R

Supplemental figure 1. Conceptual illustration of extracting positive and negative instances from each lead time group.

Supplemental figure 2. Conceptual architecture of a CNN

Supplemental figure 3. Research flow diagram

Supplemental table 1. Sample selection process

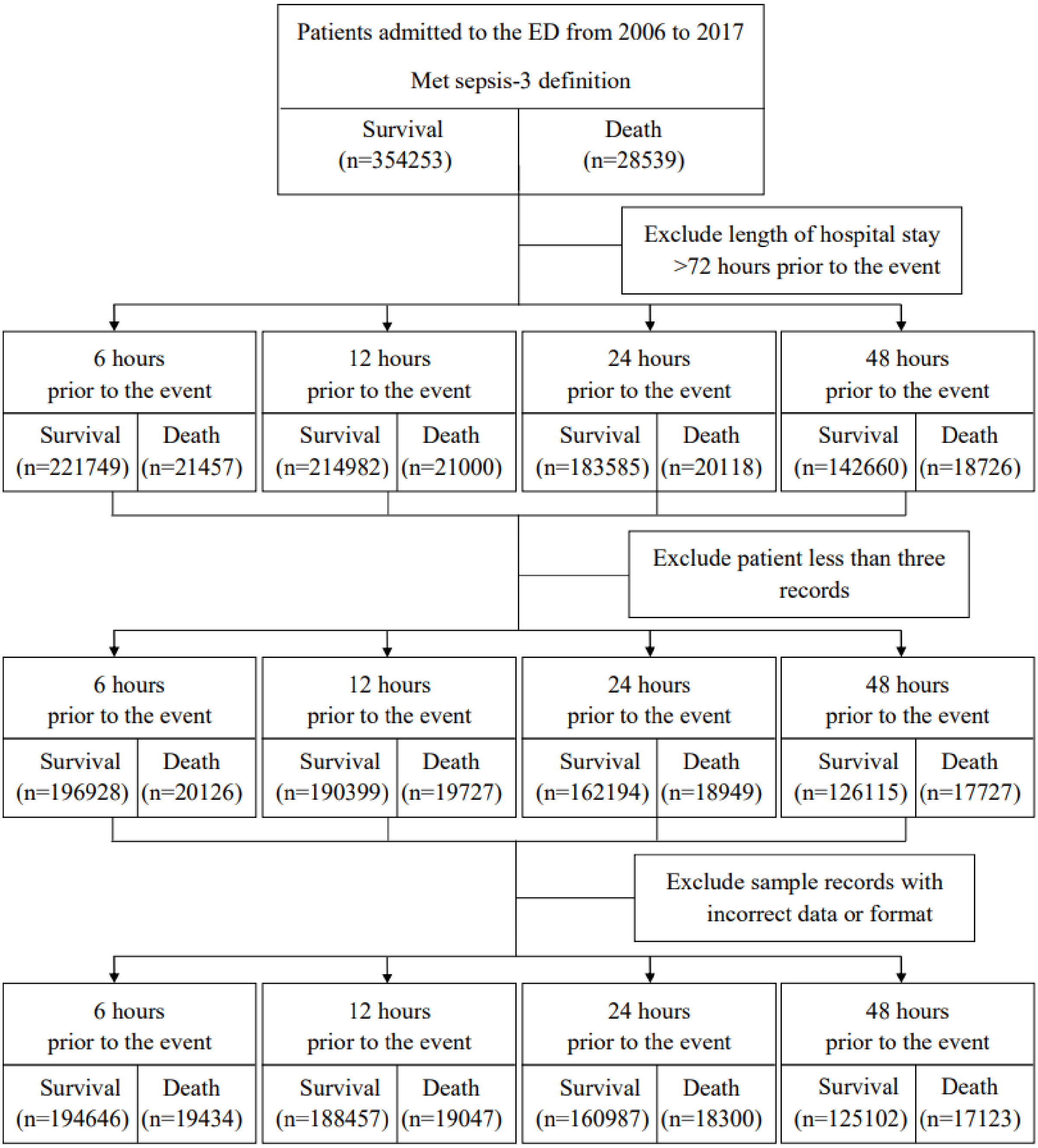

Supplemental table 2. Network architecture of CNN

**Layer name**	Components	
**Conv_1**	Filters = 1 × 3, Channels = 16, Strides = 1<br>Batch-Norm<br>ReLu	Filters = 3 × 3, Channels = 16, Strides = 1<br>Batch-Norm<br>ReLu
**Pool_1**	2 × 2 max pooling, Strides = 2	2 × 2 max pooling, Strides = 2
**Conv_2**		Filters = 3 × 3, Channels = 32, Strides = 1<br>Batch-Norm<br>ReLu
**Pool_2**		2 × 2 max pooling, Strides = [5,1]
**Concate_1**	Concatenation along an axis for filters	
**Conv_3**	Filters = 3 × 3, Channels = 96, Strides=1<br>Batch-Norm<br>ReLu	

**Pool_3**	2 × 2 max pooling, Strides = 2<br>Dropout (0.5)
**GAP_1**	Global Average Pooling<br>Softmax (2)

Supplemental table 3. Network architecture of LSTM

**Layer name**	Components
**Lstm_1**	Units = 256,<br>Kernel_init=he_uniform,<br>Activation = tanh
**Lstm_2**	Units = 12nel_init=he_uniform,<br>Activation = tanh
**Lstm_3**	Units = 128,<br>Kernel_init=he_uniform,<br>Activation = tanh
**Flatten**	Transfer results into one dimension
**Dense**	Units = 2,<br>Kernel_init=he_uniform,<br>Activation = softmax

Supplemental table 4. Parameters of Random Forest model

Parameter	value
N_estimators	80
Max_depth	No-limit (default value)
Max_features	Auto (default value)
Min_samples_leaf	1 (default value)
Min_samples_split	2 (default value)

Supplemental table 5. Training and validation results of EWS_C

	Training			Validation					
	**Precision**	**Sensitivity**	**Accuracy**	**Precision**	**Sensitivity**	**Accuracy**	**AUC**	**AUC_ COV**	**CI**
**6hr**	0.905	0.896	0.905	0.883	0.784	0.84	0.84	0.000	[0.804 0.876]
**12hr**	0.847	0.884	0.872	0.864	0.791	0.833	0.833	0.000	[0.791 0.867]
**24hr**	0.919	0.895	0.860	0.862	0.732	0.807	0.807	0.000	[0.765 0.849]
**48hr**	0.793	0.854	0.828	0.888	0.714	0.811	0.811	0.001	[0.766 0.857]

Supplemental table 6. Training and validation results of EWS_L

	Training			Validation					
	precision	Sensitivity	Accuracy	precision	Sensitivity	Accuracy	AUC	AUC_COV	CI
6hr	0.844	0.795	0.817	0.811	0.680	0.761	0.761	0	[0.719 0.802]
12hr	0.778	0.748	0.759	0.746	0.768	0.752	0.752	0.001	[0.674 0.817]
24hr	0.808	0.779	0.789	0.781	0.695	0.750	0.750	0.001	[0.703 0.800]
48hr	0.748	0.778	0.759	0.761	0.684	0.734	0.734	0.001	[0.681 0.787]

Supplemental table 7. Training and validation results of EWS_R

	Training			Validation					
	precision	Sensitivity	Accuracy	precision	Sensitivity	Accuracy	AUC	AUC_ COV	CI
6hr	0.857	0.797	0.835	0.800	0.722	0.770	0.770	0	[0.729 0.812]
12hr	0.850	0.794	0.829	0.837	0.676	0.772	0.772	0.000	[0.725 0.808]
24hr	0.837	0.776	0.810	0.816	0.701	0.771	0.771	0.001	[0.726 0.816]
48hr	0.803	0.737	0.776	0.805	0.699	0.764	0.764	0.001	[0.714 0.815]

Supplemental table 8. Cross Validation Results of EWS_C

Cross Validation Results of EWS_C (k fold = 5)			
	Precision	Sensitivity	Accuracy
**6hr**			
k fold=1	0.894	0.879	0.891
k fold=2	0.898	0.881	0.896
k fold=3	0.904	0.893	0.903
k fold=4	0.91	0.887	0.9
k fold=5	0.901	0.892	0.899
**mean**	**0.9014**	**0.8864**	**0.8978**
**95%CI**	**[0.8938,0.9089]**	**[0.8785,0.8942]**	**[0.8514 0.9442]**
**12hr**			
k fold=1	0.876	0.88	0.885
k fold=2	0.899	0.852	0.882
k fold=3	0.847	0.883	0.872
k fold=4	0.829	0.828	0.853
k fold=5	0.83	0.835	0.855
**mean**	**0.8562**	**0.8556**	**0.8694**
**95%CI**	**[0.8182,0.8941]**	**[0.8242,0.8869]**	**[0.823 0.915]**

**24hr**			
k fold=1	0.848	0.853	0.86
k fold=2	0.83	0.708	0.83
k fold=3	0.835	0.851	0.835
k fold=4	0.831	0.802	0.827
k fold=5	0.831	0.803	0.83
**mean**	**0.835**	**0.803**	**0.836**
**95%CI**	**[0.8256,0.8443]**	**[0.7303,0.8764]**	**[0.789, 0.883]**
**48hr**			
k fold=1	0.824	0.706	0.855
k fold=2	0.808	0.703	0.795
k fold=3	0.808	0.737	0.773
k fold=4	0.835	0.71	0.812
k fold=5	0.854	0.736	0.804
**mean**	**0.826**	**0.718**	**0.808**
**95%CI**	**[0.8016,0.8499]**	**[0.6976,0.7391]**	**[0.752, 0.863]**

Supplemental table 9. Cross Validation Results of EWS_L

**Cross Validation Results of EWS_L (k fold = 5)**			
	**Precision**	**Sensitivity**	**Accuracy**
**6hr**			
k fold=1	0.832	0.791	0.809
k fold=2	0.851	0.8	0.824
k fold=3	0.837	0.789	0.813
k fold=4	0.841	0.796	0.818
k fold=5	0.845	0.797	0.819
**mean**	**0.8412**	**0.7946**	**0.8166**
**95%CI**	**[0.8321,0.8502]**	**[0.7890,0.8001]**	**[0.8094, 0.8237]**
**12hr**			
k fold=1	0.798	0.832	0.83
k fold=2	0.77	0.8	0.758
k fold=3	0.748	0.839	0.812
k fold=4	0.776	0.82	0.828
k fold=5	0.801	0.805	0.838
**mean**	**0.7786**	**0.8192**	**0.8132**
**95%CI**	**[0.7515,0.8056]**	**[0.7983,0.8400]**	**[0.7731, 0.8532]**

**24hr**			
k fold=1	0.776	0.811	0.761
k fold=2	0.768	0.782	0.733
k fold=3	0.748	0.687	0.809
k fold=4	0.773	0.699	0.766
k fold=5	0.771	0.777	0.803
**mean**	**0.767**	**0.751**	**0.774**
**95%CI**	**[0.7533,0.7810]**	**[0.6830,0.8193]**	**[0.7352, 0.8135]**
**48hr**			
k fold=1	0.754	0.69	0.733
k fold=2	0.765	0.684	0.732
k fold=3	0.752	0.685	0.731
k fold=4	0.764	0.684	0.732
k fold=5	0.76	0.69	0.736
**mean**	**0.759**	**0.687**	**0.733**
**95%CI**	**[0.7535,0.7644]**	**[0.6827,0.6904]**	**[0.7304, 0.7351]**

Supplemental table 10. Cross Validation Results of EWS_R

**Cross Validation Results of EWS_R (k fold = 5)**			
	**Precision**	**Sensitivity**	**Accuracy**
**6hr**			
k fold=1	0.86	0.8	0.84
k fold=2	0.852	0.795	0.83
k fold=3	0.855	0.799	0.832
k fold=4	0.851	0.801	0.841
k fold=5	0.849	0.795	0.829
**mean**	**0.8534**	**0.798**	**0.8344**
**95%CI**	**[0.8480,0.8587]**	**[0.7944,0.8015]**	**[0.8273,0.8414]**
**12hr**			
k fold=1	0.812	0.773	0.803
k fold=2	0.844	0.781	0.784
k fold=3	0.85	0.79	0.82
k fold=4	0.831	0.807	0.825
k fold=5	0.817	0.77	0.786
**mean**	**0.8308**	**0.7842**	**0.8036**
**95%CI**	**[0.8103,0.8512]**	**[0.7656,0.8027]**	**[0.7810, 0.8270]**

**24hr**			
k fold=1	0.809	0.758	0.799
k fold=2	0.829	0.722	0.772
k fold=3	0.822	0.728	0.817
k fold=4	0.823	0.793	0.806
k fold=5	0.816	0.739	0.771
**mean**	**0.820**	**0.748**	**0.793**
**95%CI**	**[0.8103,0.8292]**	**[0.7124,0.7835]**	**[0.7673, 0.8186]**
**48hr**			
k fold=1	0.809	0.697	0.76
k fold=2	0.807	0.681	0.769
k fold=3	0.8	0.695	0.77
k fold=4	0.804	0.69	0.764
k fold=5	0.796	0.698	0.762
**mean**	**0.803**	**0.692**	**0.765**
**95%CI**	**[0.7966,0.8097]**	**[0.6835,0.7008]**	**[0.7595, 0.7704]**

Supplemental figure 1. Conceptual illustration of extracting positive and negative instances from each lead time group.

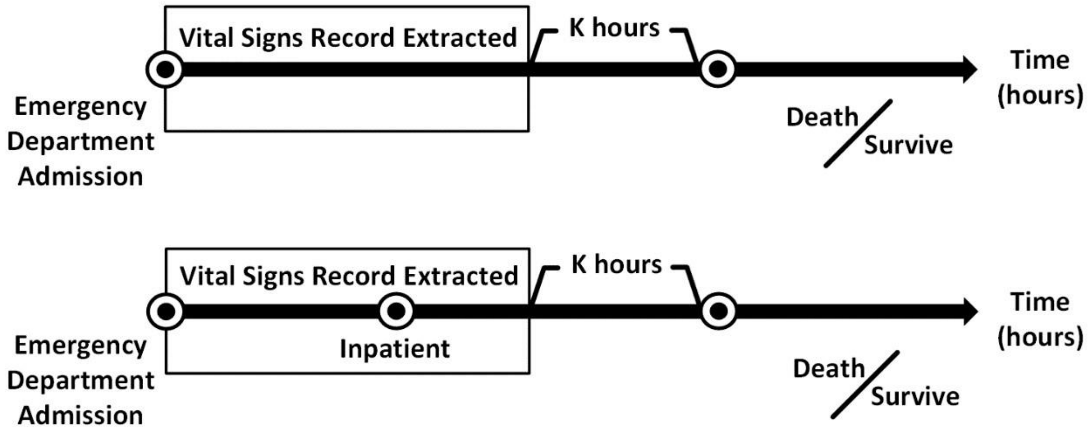

Supplemental figure 2. Conceptual architecture of a CNN

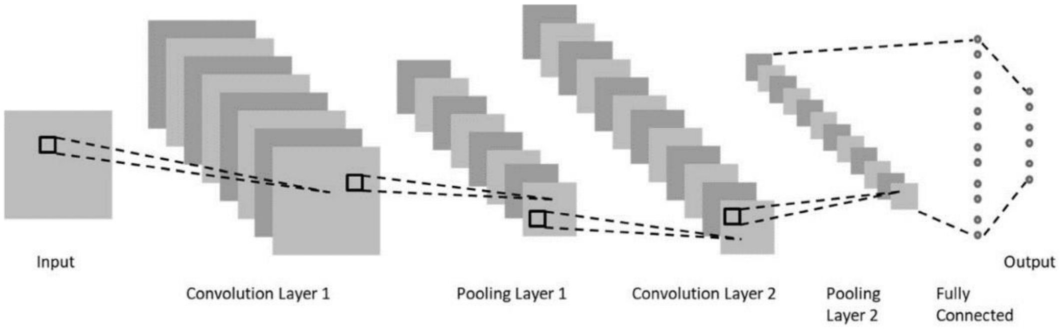

Supplemental figure 3. Research flow diagram

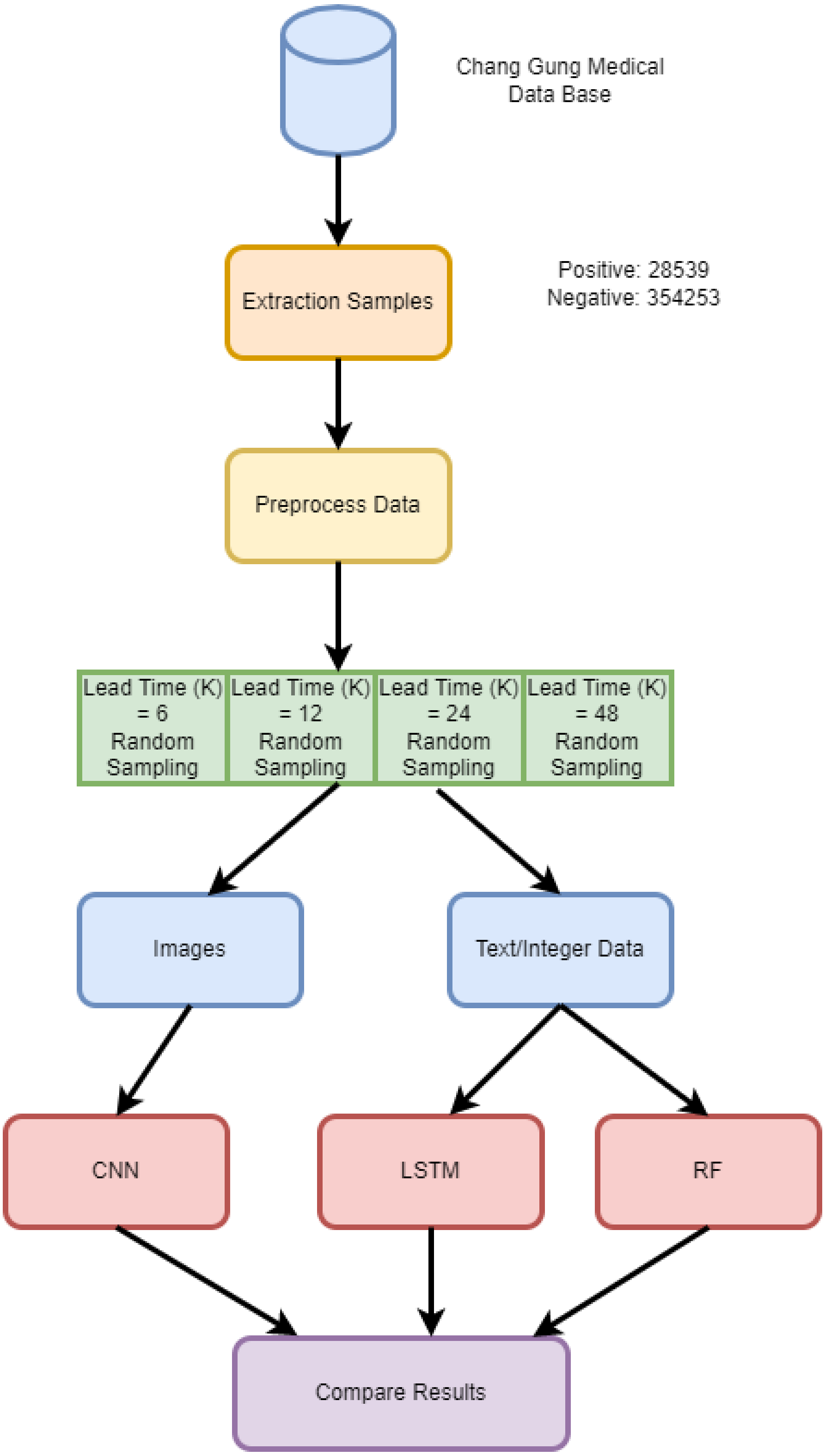

Supplement: Supplementary file 1 [file Data_Sheet_1.pdf]
